# Effects of an internationalization at home (IAH) programme on cultural awareness among medical and nursing students in Hong Kong and Indonesia during the COVID-19 pandemic: a mixed-methods study

**DOI:** 10.1186/s12909-022-03424-5

**Published:** 2022-05-13

**Authors:** Patrick Pui Kin Kor, Clare Tsz Kiu Yu, Ida Ayu Triastuti, Mitra Andini Sigilipoe, Haryo Dimasto Kristiyanto, Johana Puspasari Dwi Pratiwi, Teguh Kristian Perdamaian, Lisa Mengli Li, Phyllis Chui Ping Pang, The Maria Meiwati Widagdo

**Affiliations:** 1 Centre for Gerontological Nursing, School of Nursing, The Hong Kong Polytechnic University (School of Nursing), Hung Hom, Hong Kong; 2 University College London (Division of Psychiatry), London, UK; 3 Faculty of Medicine, Duta Wacana Christian University, Yogyakarta, Indonesia

**Keywords:** Internationalization at home, Medical education, Cultural sensitivity, Cultural awareness

## Abstract

**Background:**

The COVID-19 pandemic has severely impacted the learning experience of students by limiting their opportunities for face-to-face intercultural exchanges. Given the importance of cultural competence in medical education, there is a need to develop a programme that promotes cultural awareness, but that offers more flexibility in terms of outbound mobility. This study aims to evaluate the effectiveness of an internationalization at home programme and to explore the learning experiences of medical and nursing students from Hong Kong and Indonesia.

**Methods:**

Students were recruited from two universities in Hong Kong and Indonesia. They attended an online internationalization at home programme designed by members of the research team from both countries. A mixed-methods study was conducted using a concurrent triangulation approach.

A pre-test post-test design was used to evaluate the effects of the programme on cultural awareness, and four focus groups were conducted to explore the students’ experiences in the programme. Quantitative and qualitative data were analysed by T-test and reflexive thematic analysis, respectively. Data were integrated and triangulated using joint displays by comparing findings from both sources.

**Results:**

One hundred and forty-eight students from Hong Kong and Indonesia participated in the study. After the programme, there was a significant improvement in cultural awareness. Three themes were identified: (1) learning process: enjoyable, but a desire remains for face-to-face cross-cultural communication; (2) learning outcomes: gained cultural awareness, developed cultural sensitivity, had an opportunity to practice language and learn about new learning styles; (3) factors influencing learning outcomes: facilitators (micro-movie and active communication) and barriers (language barrier, inappropriate time arrangement, insufficient prior briefing).

**Conclusion:**

This programme achieved the learning outcomes by successfully enhancing the cultural awareness of students during a time of pandemic when outbound student exchanges were not possible. Further adaptations of the programme are required to enhance different learning outcomes.

**Supplementary Information:**

The online version contains supplementary material available at 10.1186/s12909-022-03424-5.

## Introduction

Due to globalization, the population of every country has become more culturally diverse. Healthcare professionals often need to interact with individuals whose health beliefs and life experiences are very different from their own. To meet the healthcare needs of people from different cultures, cultural competency is increasingly being regarded as an essential skill in medical training [1]. Previous studies have suggested that cultural competency may improve communication between healthcare professionals and patients, increase the satisfaction and treatment adherence of patients, and improve clinical outcomes [2].

Cultural competency can be defined as the ability to provide care to patients of diverse values, beliefs, and behaviours, including tailoring healthcare delivery to meet the demands of patients [3]. Cultural competency can be broadly conceptualized as comprised of three concepts: 1.) Awareness of one’s own culturally related biases and values, 2.) Knowledge about the cultural values of diverse populations, and 3.) Skills that can be applied when working with a culturally diverse patient group [3]. Previous studies have suggested that students can acquire cultural competency through education [1]. Students equipped with cultural competency might well be more readily prepared to accept challenges related to cultural diversity in clinical settings.

Nowadays, cultural competency training is often a component of university curricula [1]. Such training often involves providing direct multicultural experiences, such as through an overseas exchange or overseas placement. Through such face-to-face cultural exchange programmes, students can practice in settings and with cultures other than their own. However, this approach is often heavily criticized by people in the field because of its inflexibility with regard to student mobility (students must travel overseas in order to be exposed to this education). Not only is this approach inherently inequitable for the financially disadvantaged, but this type of programme also fails completely when border restrictions are in place, such as during the Covid-19 pandemic. Hence, there is a need for an internationalization at home programme (IAH) [4, 5].

The concept of Internationalization at home (IAH) was first proposed at the 1999 spring forum of the European Association for International Education’s (EAIE). It was simply defined as ‘any internationally related activity with the exception of outbound student and staff mobility’ [6]. It was later argued that the concept of IAH is not an aim or a didactic concept in itself, but rather a set of teaching and learning activities conducted ‘at home’ with the aim of developing the international and intercultural competencies of students [7]. A newer concept of IAH has been proposed, which stresses the intentional inclusion of international and intercultural aspects into curricula in a purposeful way. Specifically, it is ‘the purposeful integration of international and intercultural dimensions into the formal and informal curriculum for all students within a domestic learning environment’ [7]. IAH can also serve as a way to enhance common beliefs and bring about a closer understanding between people of different cultural backgrounds, enhance cooperation between institutions of higher education in their internationalization efforts, and improve teaching quality through mutual learning, comparisons, and exchanges of advice on good practices [8].

IAH has been implemented by several institutions of higher education around the world such as Sweden, the UK, Germany, Australia, South Africa, and China [7]. It is also a key priority area of the European Commission’s policy for international cooperation in education and training with non EU-countries [8]. Currently, more and more European institutions of higher education are embracing IAH as an institutional policy for internationalizing their curricula, teaching and learning activities, and extra-curricular activities, as well as for liaising with local cultural and ethnic groups involving different international partners. For example, 31 nursing students from Sweden, Australia, and Hong Kong participated in a series of IAH programmes in 2017. The study showed that the students who had participated in the IAH activities became aware of their own professional and personal ethical ideals, and improved their relational skills. IAH activities have also been helpful in empowering students to take the lead and encourage each other to value diversity [9].

On the other hand, a scoping review of the internationalization of medical education in 2020 revealed that although there has been a drastic increase in research in this area in the last 25 years, the number of published peer-reviewed scientific articles on this subject has been limited. In this review, significant findings were limited because only a few medical schools have engaged in IAH, and comparative data from related professions are lacking, with some schools and professions not focusing on standardized goals [10]. Furthermore, most existing internationalization programmes for nursing/medical students have focused on clinical students, who usually have much more medical knowledge and experience, and not on preclinical students [11]. Although international exposure can have lifelong effects on cultural competency, cultural competence is a multi-dimensional construct that includes a person’s cultural sensitivity, attitudes, cultural awareness, and cultural knowledge and skills, all of which take time to develop [12]. The early implementation of IAH on preclinical students can help them to develop their intercultural skills at an early stage and maintain them in later years.

Considering the on-going pandemic and the limited opportunities for physical mobility, IAH has a stronger role than ever to play in empowering cultural competencies. There is an imminent need for effective IAH programmes that allow students to develop intercultural competencies through a virtual platform [13]. The IAH programmes should also be promoted in the future to address the needs of different students, such as those who are too financially disadvantaged to travel abroad. Although IAH has been studied for more than a decade, a recent scoping review of IAH indicated that most publications on IAH were only qualitative studies exploring the learning experiences of students or case studies without a rigorous research design [14]. To the best of our understanding, our study is the first to investigate the effects of IAH on cultural awareness in medical education using a mixed-methods design. The qualitative and quantitative findings, as well as the integration of the data, give us a better understanding of the adaptation of IAH in medical education. Also, we delivered IAH to students in the health professions, with robust technology assisting in the preparation, class sessions, and evaluation of the programme in this study.

This study aims to evaluate the effectiveness of the internationalization at home programme on cultural awareness among medical and nursing students; and to explore the learning experiences of medical and nursing students from Hong Kong and Indonesia. The objectives of this study were as follows: 1.) To evaluate the effectiveness of an IAH programme in raising the cultural awareness of the students, 2.) To explore the students’ experiences with the IAH programme, and 3.) To determine facilitators and barriers to the use of the programme.

## Methods

### Design

This study adopted a concurrent mixed-methods design, which involved conducting surveys and focus groups. This approach was chosen because of our multiple sources of data, which were both quantitative (i.e., levels of cultural awareness) and qualitative (i.e., experiences, facilitators, barriers) in nature. In addition, the data integration in the mixed-methods design linked the two approaches and dimensions together to create a new and more holistic understanding (e.g., convergence and divergence) than could be achieved by either of the approaches alone, and was an advantage when evaluating our relatively new IAH programme [15, 16]. Both types of data have equal value for understanding the impact of IAH and can be used to corroborate and expand on each other, so the current approach was adopted. For the quantitative arm, we used a pre−/post-test design to investigate the students’ cultural awareness after the programme, while the focus groups were conducted to explore the experiences of students in the programme. We collected the quantitative data (post-test) and qualitative data simultaneously and independently, immediately after the programme (within 1 week after the programme). The ethics committee of The Hong Kong Polytechnic University (HSEARS20210604001) approved this study.

### Study participants and setting

The participants were nursing students from the School of Nursing, The Hong Kong Polytechnic University and medical students from the Duta Wacana Christian University in Indonesia. Students were recruited from a subject called ‘Healthy Lifestyle Challenges for Developing Communities’. This course is an elective at both universities. All of the activities were conducted via the online platform ZOOM.

### IAH programme

The IAH programme was developed by the four nursing and medical academics (PK, PP, TW, IT) who each have over 15 years of experience in clinical education and curriculum development. The project proposal, including the programme, was also reviewed by a group of experts in IAH and clinical education from the Hong Kong Polytechnic University for the awarding of a teaching grant. The programme was developed following the principles of IAH identified by Crowther et al. (2000), namely: (1) diversity as a resource, (2) an internationalized curriculum, and (3) a culturally sensitive pedagogy [6]:

1. Diversity as a resource
  We adopted cultural diversity as a resource in integrating the experiences and knowledge of both PolyU and UKDW students from diverse backgrounds in the teaching and discussion.
2. An internationalized curriculum
  We adopted an internationalized curriculum in the subjects, teaching students how local and global contexts intersect in managing health-related problems (see the subject objectives below)
3. Culturally sensitive pedagogy
  Enquiry-based learning was adopted to build empathy among the PolyU and UKDW students, towards people in different situations from themselves. The students were asked to watch a micro-movie simulating the life of an Indonesian domestic helper living in Hong Kong.

To align with the subject objectives in promoting a healthy lifestyle, the subject team drew up the following expected outcomes. After the IAH programme, students will be able to:

1. examine how local and global contexts intersect in managing health-related problems, which include unhealthy eating habits, a lack of physical exercise, and addictive behaviours (e.g., smoking);
2. apply evidence, concepts, and theories to problem cases related to leading an unhealthy lifestyle in different cultural contexts;
3. develop a cross-cultural understanding of the social and cultural determinants of a healthy lifestyle;
4. broaden their international and cross-cultural awareness and intercultural sensitivity;
5. enrich their preparedness in the areas of social responsibility and a global outlook.

Five sessions of an online IAH programme (4 hours each) were provided for the PolyU students and the Indonesian students from Duta Wacana Christian University. To align with the above outcomes, we designed the following teaching and learning activities:

1. To understand how local and global contexts intersect in managing health-related problems, which include unhealthy eating habits, a lack of physical exercise, and addictive behaviours; and to enrich the students’ preparedness in the areas of social responsibility and a global outlook
  An enquiry-based learning (EBL) approach was adopted. A micro-movie was developed to simulate the life of an Indonesian domestic helper in Hong Kong who looks after a Chinese older person. The video portrayed an Indonesian lady living in Hong Kong who helps to take care of an older person. Both the Indonesian lady and the older person had various unhealthy lifestyle habits. For example, the Indonesian lady always eats fast food and junk food, and the older person does not engage in sufficient physical activity. Also, the Indonesian lady faced various difficulties when providing care for the older person with regard to the appropriate diet and types and amount of physical activity because of her lack of knowledge of the needs of older people and cultural differences. In this workshop, students from different cultural backgrounds were asked to form small groups. Together, they searched for and used various resources to address the above problems. The students had to determine what they needed to learn, identify appropriate learning resources, report their learning outcomes, and assess their progress in learning. Through this learning process, the students were expected to understand more about how the same health problem is managed in two different regions among people of different cultural backgrounds, Chinese and Indonesian. During the discussion, the teacher also guided the students to further understand how local and global contexts intersect in managing health-related problems.
2. To apply evidence, concepts, and theories to problem cases related to leading an unhealthy lifestyle in different cultural contexts; and to develop into a socially responsible global citizen
  Before the IAH programme, the students had already learnt about health assessment skills, health promotion strategies, global citizenship, and intercultural competence from the lectures and in-class activities. In the programme, the students worked together to develop a health promotion plan for the cases in the micro-movies. The students did not only apply the skills that they had learnt from the lectures, but also needed to learn and discuss with the foreign students about cultural contexts when developing a health promotion plan for the Indonesian and Chinese people. The students had to make a group presentation during the workshop and the different student groups had to critically appraise the health promotion plan developed by the respective peer groups.
3. To develop a cross-cultural understanding of the social and cultural determinants of a healthy lifestyle and to broaden the students’ cross-cultural awareness and intercultural sensitivity
  This programme started with an ice-breaking activity by students from the two universities, which involved introducing different traditional foods and lifestyles in Hong Kong and Indonesia, followed by a discussion about their implications for a healthy lifestyle. An e-library was also set up to keep a record of various open-source teaching materials (e.g., website, videos, news) about health problems relating to unhealthy eating, a lack of physical exercise, and additive behaviours in Indonesia and Hong Kong. The students were asked to read those pre-workshop materials in the e-library to enhance their social and cultural understanding of a healthy lifestyle.

### Data collection

#### Quantitative survey method

Convenience sampling was adopted for subject recruitment. All of the students who were enrolled in the subject ‘Healthy Lifestyle Challenges for Developing Communities’ in the study cohort 2020/2021 were invited to join the study through an email sent by an independent research assistant. The Qualtrics XM Online Surveys Platform was adopted to collect their consent to participate and their quantitative data. Their participation in this study was entirely voluntary and anonymous. Since the IAH programme was newly developed for this study, to estimate the size of our sample we adopted a conservative effect size of 0.45 (medium effect) obtained from a meta-analysis of educational interventions for promoting cultural awareness [17]. Considering the 20% attrition rate, a sample size of 96 students would be needed to detect the effects of the intervention on cultural awareness at a power of 80% and a two-sided significance level of 5%. Informed consent was received from all of the participants before the commencement of data collection. The students were asked to complete an online survey 1 week before (T0) and after (T1) the programme. The survey includes the modified cultural awareness scale [18]. The modified CAS was used to measure the cultural awareness (minimal level of cultural competence) of students in higher education. The scale contains a total of 35 items. The responses are based on a 7-point scale (strongly disagree to strongly agree, with a midpoint choice of no opinion) and one additional response of ‘does not apply’. This scale generates four subscale scores: ‘General educational experience (ranging from 15-105)’, ‘cognitive awareness (ranging from 7-49)’, ‘clinical issues (ranging from 5-35)’, and ‘behaviours/comfort with interactions (ranging from 8-56)’. The total score ranges from 35 to 245. A higher score indicates greater cultural awareness. The scale was demonstrated to have satisfactory validity, with a Kaiser-Meyer-Olkin value of 0.738, and satisfactory reliability, with a Cronbach’s alpha value of 0.88, among tertiary students from Sweden and Hong Kong in healthcare fields [18].. Demographic data, including age and gender, were also collected at baseline (T0). The data were analysed using IBM’s SPSS (version 25). A paired sample T-test was conducted, and a two-sided significance test (*p* < .05) was applied.

#### Qualitative focus group method

An online consent form was received from all of the participants before the focus groups were conducted. Purposive sampling was used to select participants with a range of characteristics that might be related to their learning experience, such as study major and year of study. Four semi-structured focus groups were conducted to understand the participants’ perceptions of and experiences with the online IAH programme. Hong Kong and Indonesian students were part of separate focus groups. Each focus group was led by three instructors (one Hong Kong and two Indonesian) with prior knowledge of the internationalization at home pedagogy and rich experience in conducting focus groups. A research assistant helped to take notes in each focus group. In the focus group, a guiding question was first employed to open up new testimonies: ‘What was your learning experience in taking the online IAH programme?’ The participants were then asked about how the experience impacted their study, the elements that they liked/disliked, and the skills, knowledge, and any other benefits that they had gained.

All of the focus groups were audio-recorded on ZOOM and the proceedings transcribed and translated into English. Interview data were inductively analysed using Braun and Clark’s reflexive thematic analysis approach [19, 20]. Initially, two researchers (PK and CY) independently undertook the task of repeatedly reading and listening to the transcripts and interview recordings. After conducting double-coding and collaborative discussions, initial codes were sorted into themes. The researchers analysed the data until the point of data saturation was reached, when no new findings emerged. After this, an initial coding framework was generated, which was reviewed by a third researcher (IA). The final coding framework was then developed through on-going discussions and by continuously refining and defining themes. Then, the first author (PK) wrote up a report analysing the results, which was circulated to all of the authors and approved by them. The researchers (PK, CY, and IA) who participated in the thematic analysis reflected that their own knowledge and experiences might have influenced the analysis. Throughout the process, disagreements were resolved through discussions between PK and CY.

To enhance the trustworthiness of the qualitative data, the Lincoln and Guba criteria were adopted in this study namely credibility, dependability, confirmability, and transferability [21]. The criterion of credibility was obtained through a peer review and by reviewing the texts of the interviews of the participants. The criterion of dependability was met by the careful keeping of data records, rich descriptions, and responsiveness, meaning that an external supervisor familiar with qualitative research examined the research process and data analysis. Confirmability of data was achieved by having at least two experts in the field of qualitative research and international health education review the reports and writings. The transferability criterion was met through writing about the whole research process in detail.

### Data integration

Quantitative and qualitative data were analysed as described above. Both types of data were integrated through triangulation [22]. Through the data triangulation process, we compared the qualitative and quantitative findings by tabulating the themes and survey findings (a process of joint displays). The areas of divergence and convergence were discussed iteratively by CY and PK. These synthesized concepts were then used to understand the learning experience of students in the IAH programme. To guarantee the rigour of this mixed-methods study, we followed legitimation criteria (Commensurability approximation legitimation, Inside-outside legitimation, Integration legitimation, Paradigmatic legitimation, Sample integration legitimation, Sequential legitimation, Socio-political legitimation, Weakness minimisation legitimation) [23]. A full description of each criterion is reported in Supplementary Table 1.

## Results

### Participants

A total of 150 students enrolled in the programme. Of these, 148 (98.6%) completed four sessions, and 148 completed the pre-test post-test survey. The demographic characteristics of our participants are summarized in Table 1. Characteristics of the participants. Of the students, 67.6% (*n* = 100) were from Indonesia and 33.1% (*n* = 48) from Hong Kong. The majority were females (62.8%), and their mean age was 21 years (SD = 1.39 years). Most (*n* = 99, 83.9%) were undergraduate students from the discipline of health sciences (i.e., Nursing, Medicine).

**Table 1:** Participant’s characteristics (*N* = 148)

Age *(M, SD)*	*(21.44, 1.39)*	
	N	(%)
Gender		
Female	93	62.8
Male	55	37.2
Country		
Hong Kong	48	32.4
Indonesian	100	67.6
Have you ever visited to Indonesia before?^a^		
Yes	3	6.3
No	45	93.8
Have you ever visited to Hong Kong before^b^		
Yes	3	3.0
No	97	97.0
Major of Study		
Applied Science	1	0.7
Computer Science	1	0.7
Engineering	1	0.7
Fashion and Textiles	1	0.7
Health Sciences (Nursing, Medicine)	129	87.1
Others	15	10.1
Year of study		
Year 1	22	14.9
Year 2	19	12.2
Year 3	7	4.7
Year 4	98	66.2
Year 5 or above	2	1.4
Do you have any experience in joining cultural exchange/cultural awareness’ workshop/training?		
Yes	8	5.4
No	140	94.6

^a^This question was only answered by students from Hong Kong

^b^This question was only answered by students from Indonesia

The majority of our participants (over 93%) had never visited the partner country/region (Indonesia/Hong Kong). Only 5.4% (*n* = 8) had attended a cultural exchange programme before, suggesting that our participants from Hong Kong and Indonesia had limited exposure to each other’s culture.

### Cultural awareness

The result of the programme’s impact on raising cultural awareness is summarized in Table 2 Results of a paired T-test comparing cultural awareness scores before and after the workshop. Our result indicates that after the programme, there was a significant increase in all of the subscale scores on cultural awareness [General education & research experience (t(60) = − 3.23, *p* = .00), behaviours/comfort with interactions (t (55) = − 2.72, *p* = .01), cognitive awareness t(65) = − 2.22, *p* = .03), clinical issues t(67) = − 3.01, *p* = .00)], and in the total score (t(51) = − 3.23, p = .00).

**Table 2:** Results of paired T-test comparing cultural awareness score before and after the workshop

	Pre-test score		Post-test score		*df*	*t value*	*p value*
	*M*	*(SD)*	*M*	*(SD)*			
General education & research experience	74.10	11.07	80.39	13.85	60.00	−3.23	< 0.001
Behaviors/comfort with interactions	31.70	7.58	35.77	9.70	55.00	−2.72	0.01
Cognitive Awareness	35.00	5.02	36.89	5.90	65.00	−2.22	0.03
Clinical issues	25.96	4.31	28.12	4.78	67.00	−3.01	< 0.001
Cultural awareness score total score	164.67	19.03	179.63	29.36	51.00	−3.23	< 0.001

Remarks: The cultural awareness sale (CAS)generated four subscale scores. This includes general educational experience (subscale score ranges from 15–105),behaviors/comfort with interactions (subscale score ranges from 8–56), cognitive awareness (subscale score ranges from 7–49), clinical issues (subscale score ranges from 5–35). It also generates a total score ranging from 35 to 245, with higher score indicating higher cultural awareness

### Qualitative findings

A total of 16 students were interviewed in four semi-structured focus group interviews (two each for the Hong Kong and Indonesian students). We identified three major themes in our analysis of the data and present these in each subsection below. The first theme ‘learning process’ describes students’ perceptions and feelings towards the programme. The second theme, ‘learning outcomes’ describes what outcomes (culture-related and non-culture related) students believed they had achieved from the programme. The third theme, ‘factors influencing learning outcomes’ describes factors that facilitate and hinder the learning process and learning outcomes. Together, these themes provide context to the general learning experience of the programme. Throughout the analysis, we aimed to provide a sense of the contents of the data by using quotes from anonymized participants, whose country of origin was indicated using the prefix of IN (students from Indonesia) or HK (Hong Kong). Details are stated as follows:

#### Learning experience

1. **IAH as an enjoyable experience**

Both the students from Indonesia and Hong Kong described the IAH experience as ‘fun’, ‘interesting’, and ‘exciting’. These descriptions often came with coding related to a ‘novel experience’. Specifically, many students said that this programme enabled them to gain new knowledge of another culture and meet students from another country, and that is what excited them the most.

*‘It is interesting to discuss culture with people from other countries. In my opinion, it is very interesting. It helps us to widen our horizons and allows us to understand that everyone is different.’ (HK-GP1)*

*‘I am excited. I think cultural exchange is an interesting thing. I grasped every opportunity for discussion so that I could get to know them (Hong Kong students) better. I mean … I was given these opportunities. So, why would I not use these opportunities properly? I feel happy and excited because I can meet people from a different background.’ (IN-GP 1*)

*‘I am really excited because it is the first time that I have gotten a chance to collaborate with foreigners –the Hong Kong students. That teaches me how to work with new people.’ (IN-GP 2)*

2. **Viewing IAH as a novel experience, but still valuing the essence of face-to-face interactions**

Although students generally appreciated the novelty of the IAH experience and the convenience of not having to travel abroad, in the focus groups the students constantly compared the online cultural exchange experience with face-to-face cultural exchange activities. Most indicated that they would still choose face-to-face interactions over an online cultural experience if offered both options.

*‘I still prefer to know more about the culture by visiting there (Indonesia) locally. That was my goal. I mean … if I were given the chance to taste the local food there, it would be better for me to learn about their culture.’ (HK-GP 2)*

*‘This programme allows us to learn about the health conditions of other countries even if we don’t have to go on a trip. We can still widen our horizons.’ (IN-GP 2)*

Interestingly, one student described the important role of IAH during the COVID-19 pandemic, when all face-to-face cultural exchange activities were suspended. ‘*Because of the pandemic, there is no chance to go abroad, so this programme does give us a chance to communicate with students from other countries. It is better than nothing.’ (IN-GP 2).*

#### Learning outcomes

This theme features learning outcomes that the students believed they had achieved from the programme.

1. ***Awareness of cultural diversity (new knowledge, noticing differences, social responsibility)***

Many students said that they gained awareness of cultural diversity after attending the programme. Students developed such awareness through gaining knowledge about the health habits and health policies of another country. Such awareness often came as a surprise to the students. One student from Indonesia talked about her awareness of cultural diversity in alcohol addiction. *‘My group was responsible for the topic of addiction. So we discussed the cultural reasons behind alcohol abuse. Like related laws and regulations. I am surprised that alcohol is available everywhere in Hong Kong and that it is even available in vending machines. The drinking habit there is deeply embedded. For example, alcohol is often served in their company events for the purpose of social bonding, or in family events, so I can imagine that it is harder to manage in Hong Kong.’* An Indonesian student also mentioned that she realized there were more informational resources available in Hong Kong. *‘They (HK-GP2) have a variety of resources – different educational materials and databases. Here in Indonesia, I think we only have materials from the Health Ministry. When the Health Ministry says something, we always follow along. But they have more than that, and this information is helpful to our project.’ (IN-GP1).*

Interestingly, from the focus groups with students, we realized the new knowledge was not simply coming from interactions within the programme (i.e., discussions, presentations). The students gained new knowledge about another country from social interactions as well, which occurred outside the classroom. For example, a student from Indonesia said that she connected with one of her group mates from Hong Kong via Instagram (a social media platform). *‘We read each other’s Instagram stories. And I can see that from her story, she was hanging out with her girlfriends and her boyfriend. This is a good exchange. You can learn about their lifestyle, just like health. It really made me become more curious about the Hong Kong culture.’ (IN- GP 1).*

By raising their cultural awareness, a few students expressed the view that they further developed a sense of international social responsibility. *‘I gained more understanding of the health situation in another country, and we learned to view health problems not just from a Hong Konger’s perspective. I have learned to care about the health issues of other people around the world. Some places require attention from us.’ (HK-GP1)* A student from Indonesia said, *‘It widens our horizons and allows us to understand that everyone is different. At some point, I realized that we have an obligation to help them. We should respect them. You gained insights and thoughts.’ (IN-GP2).*

2. ***Developed cultural sensitivity in clinical practice***

Many students highlighted the view that after attending the programme, they started to realize the importance of cultural sensitivity in clinical practice. Through the activities, especially the group discussions, they learned that cultural sensitivity is always needed when analysing health problems and that cultural adjustments are needed when developing health promotion strategies.

They also said that if communication is not culturally sensitive, it could potentially negatively impact the care provided to patients.

*‘I also feel that it is very important for us to learn about culture, because it will greatly affect the way we communicate with patients and the management of health services.’ (INDO-GP2)*

3. ***Trained up language skills***

Some students mentioned that although they might not speak English well, IAH provided a precious opportunity to practise their English.

*Many of our local students communicate in Cantonese, but in order for us to interact with the Indonesian students, we cannot use our mother tongue. Our English level may not be good enough. I mean, when you want to express your ideas, you may not be able to articulate well. In the end, we have to use English to explain ourselves. I was able to practise my spoken English. (HK-GP1)*

*‘It is very interesting, speaking English, listening, and learning, and again to talk to foreigners. Yes, in my opinion I gained additional knowledge.’ (INDO-GP1)*

4. ***Adoption of new learning styles***

Some students said that through interactions with students from another country, they learned to model their learning styles and adopted some practices, such as viewing health issues from another perspective, being more proactive in group discussions, and engaging in teamwork.

*‘I am only a year 1 student. The Indonesian students were able to think of more aspects and perspectives than me. I learned to view things in a different perspective.’ (HK-GP1)*

*‘The first thing I noticed is that students from Indonesia really took the initiative to speak up, and they are more proactive than our Hong Kong students, so I started to be more proactive as well in the discussions.’ (HK-GP1)*

*‘I have learned the importance of a team in the two-day workshop on internationalization.’ (INDO-GP1)*

#### Factors influencing learning outcomes

1. ***The micro-movie as a good teaching tool to stimulate discussion***

Students constantly brought up the micro-movie as a good teaching tool, as they thought that the movie was ‘interesting’ *(INDO-GP2)* and ‘stimulating’. They also thought that, in general, it does a good job of depicting cultural differences between Hong Kong and Indonesia.

*‘The main characters in the movie are the Hong Kong lady and the Indonesian mom. There are a lot of conflicts in their lifestyles. The conflict was especially obvious when both characters lived together…. For example, the mum doesn’t eat luncheon meat. It’s because there is so much oil, salt, sugar, and so on in it. So, I think the movie captured the difference in eating habits well.’ (IN-GP2)*

*‘There was a discussion after the movie. The movie stimulated our discussion. It's enough, to stimulate our discussion about diet, physical activity, addiction. We already know what will be discussed.’ (HK-GP1)*

Despite this, some students suggested that the micro-movie should place an equal focus on the cultures of Hong Kong and Indonesia. An Indonesian student said, *‘The characteristics of Indonesia are not outstanding enough in the micro-movie. For example, after I watched the video, I knew that there were some cultural gaps and cultural differences, but I don’t really know what the mom (Indonesian) was not familiar with … I mean specifically what aspects…. It would be better if the storyline focused more on the mom.’ (IN-GP1).*

2. ***Active communication as a factor in facilitating cultural exchanges***

Some students mentioned that active communication between group members is a key factor in facilitating discussions about culture. *‘For my group, the communication is really good…. All of them are considered active in my opinion. This really helps with the discussion.’ (IN).*

Although active interactions were described as a key factor in cultural exchanges, the language barrier was often mentioned as a factor that hindered such interactions. Many students said that there was some awkwardness in their group discussions, as some group members were reluctant to speak English. *‘In my group, the problem is more that they don’t want to communicate in English … students from both countries. It is very awkward.’ (IN-GP2) ‘There are still people at our university who are still not confident in speaking English, even though they are able to do it, but they are shy. (IN-GP1).* A few students suggested that further breaking the groups into smaller groups might facilitate more active discussions and interactions. (IN-GP2).

3. ***Time management***

Many students mentioned that the time management of the workshop could be improved. They held different opinions about the duration of the activities, with some suggesting that some activities should be longer and some shorter. Most students said that there was limited time (1 week) to prepare for the group project presentation, as online collaboration with group members from another country requires extra effort to coordinate.

*‘Personally, it has something to do with the time, the project time. I think the time is too short to work on a project between students of two countries.’ (IN-GP2)*

As almost all of the Indonesian students were in their final year, many interviewees from Indonesia expressed the view that the programme schedule didn’t fit well into their busy schedule. They thought that the cultural exchange workshop should target junior year students rather than senior year students. *‘This workshop should not be placed in the senior year, as it clashes with our thesis and community services…. If possible, it should be placed in the second or third year.’ (INDO- GP1).*

4. ***Briefing prior to the workshop***

Some students said they were not well briefed prior to the workshop, so that what they experienced in the workshop was different from what they had expected. Some students expressed disappointment. They said that one improvement would be to give the students a short briefing / workshop briefing note prior to the workshop.

*‘I thought it was a student exchange programme. But that was different from my initial expectation. It turned out to be a programme for learning each other's culture.’ (IN- GP1)*

*‘The overview for this event was a bit sudden (referring to the overview note that the team sent to students prior to the workshop). We didn’t know what this event was about…. The downside would be the short notice beforehand.’ (IN-GP1)*

### Data integration

Table 3 illustrates the data integration and meta-inference through the joint display of quantitative and qualitative data [24, 25]. First, we found that the results from the quantitative arm mostly converged with the findings from the qualitative interviews. Specifically, there was an improvement in the cultural awareness total score and in the subscales scores on ‘cognitive awareness’ and ‘clinical issues. In line with this, in our qualitative interviews, participants said that they developed cultural awareness through gaining knowledge about other countries (e.g., health habits, policies, learning styles of students), and that this is also helping them to become more culturally sensitive in clinical practice. Second, our qualitative findings provided context for the quantitative results in that they touched on the facilitators and barriers to achieving the intended learning outcomes of our programme. We found that having an enjoyable and novel experience in the virtual classroom is a key factor in the successful implementation of the programme. Using the micro-movie to stimulate active discussions further enhanced the learning experience, while language barriers were identified as an obstacle. Third, we identified areas of divergence between the quantitative and qualitative data. Our qualitative findings revealed themes that had not emerged in our quantitative findings. Some students revealed that they gained other skills (apart from cultural awareness) from the programme, such as language skills. Some students also raised concerns about the logistics of the programme, such as poor time management and insufficient briefing.

**Table 3:** Joint displays of quantitative data and qualitative data

Concept	Meta-inferences	Quantitative results		Qualitative findings	
		Inferences of quantitative findings	Quantitative results	Inferences of qualitative findings	Qualitative findings verbatims
**Learning process - IAH as an enjoyable experience**	The qualitative findings provide a context for the learning process in IAH program and explain what leads to the positive result in quantitative. (Convergent)	After the IAH program, students feel more comfortable in interacting with people from different cultural background, and students become more satisfied about the multicultural learning of the university	Significant increase in CAS score on subscales ‘Behaviours/comfort with interactions’ and ‘general education and research experience ‘	IAH as an enjoyable experience	*“It is interesting to discuss culture with people from other countries. In my opinion, it is very interesting. It helps us to widen our horizons and allows us to understand that everyone is different.’ (HK-GP1)* *‘I am excited. I think cultural exchange is an interesting thing. I grasped every opportunity for discussion so that I could get to know them (Hong Kong students) better. I mean … I was given these opportunities. So, why would I not use these opportunities properly? I feel happy and excited because I can meet people from a different background.’ (IN-GP1*) *‘I am really excited because it is the first time that I have gotten a chance to collaborate with foreigners –the Hong Kong students. That teaches me how to work with new people.’ (IN-GP2)*
**Learning process -Viewing the experience as a new form of cultural exchange, but still valuing the essence of face-to-face interactions**	The qualitative findings provide a context for the learning process in IAH program and explain what leads to the positive result in quantitative. (Convergent)	After the IAH program, students feel more comfortable in interacting with people from different cultural background	Significant increase in CAS score on ‘Behaviours/comfort with interactions’	Viewing the experience as a new form of cultural exchange, but still valuing the essence of face-to-face interactions	*‘I still prefer to know more about the culture by visiting there (Indonesia) locally. That was my goal. I mean … if I were given the chance to taste the local food there, it would be better for me to learn about their culture.’ (HK-GP2)* ‘*Because of the pandemic, there is no chance to go abroad, so this programme does give us a chance to communicate with students from other countries. It is better than nothing.’ (IN-GP2)*
**Facilitators - The micro-movie as a useful medium for triggering cross-cultural discussions and interactions**	The qualitative findings explains the quantitative findings, allowing us to understand what facilitates a more comfortable interaction with students from other country -Micro movie triggers their discussions in the IAH session. (Convergent)	After the IAH program, students feel more comfortable in interacting with people from different cultural background Students become more satisfied about the multicultural learning of the university	Significant increase in CAS score on subscales ‘Behaviours/comfort with interactions’ and ‘general education and research experience ‘	The micro-movie facilitates cultural discussions	‘*The main characters in the movie are the Hong Kong lady and the Indonesian mom. There are a lot of conflicts in their lifestyles. The conflict was especially obvious when both characters lived together…. For example, the mum doesn’t eat luncheon meat. It’s because there is so much oil, salt, sugar, and so on in it. So, I think the movie captured the difference in eating habits well.’ (IN-GP2-Micromovie)* *‘There was a discussion after the movie. The movie stimulated our discussion. It’s enough, to stimulate our discussion about diet, physical activity, addiction. We already know what will be discussed.’ (HK-GP1-Micromovie)*
**Facilitators - Active discussion as a facilitator of successful cultural exchange**	The qualitative findings can explain the quantitative findings, allowing us to understand what facilitates a more comfortable interaction with students from other country- Active discussion (Convergent)	After the IAH program, students feel more comfortable in interacting with people from different cultural background	Significant increase in CAS score on subscales ‘Behaviours/comfort with interactions’	-Active discussion leads to a successful cultural exchange -Language barriers lead to an inactive discussion	*For my group, the communication is really good…. All of them are considered active in my opinion. This really helps with the discussion.’ (INDO-Active communication)* *‘In my group, the problem is more that they don’t want to communicate in English … students from both countries. It is very awkward.’ (IN-GP2) ‘There are still people at our university who are still not confident in speaking English, even though they are able to do it, but they are shy. (IN-GP1-Active communication).*
**Learning outcome - Awareness of cultural diversity (new knowledge, noticing differences, social responsibility)**	The qualitative findings provides a context for quantitative findings and explain why cultural awareness is enhanced- Cultural awareness is enhanced by learning new knowledge and noticing differences and similarities in cultures (Convergent)	After the IAH programme, students started to aware more about the impact of cultural value on belief, attitudes, and behaviors.	Significant increase in CAS score on subscale ‘cognitive awareness’	Awareness of cultural diversity via gaining new knowledge and noticing differences and similarities in cultures	*‘My group was responsible for the topic of addiction. So we discussed the cultural reasons behind alcohol abuse. Like related laws and regulations. I am surprised that alcohol is available everywhere in Hong Kong and that it is even available in vending machines. The drinking habit there is deeply embedded. For example, alcohol is often served in their company events for the purpose of social bonding, or in family events, so I can imagine that it is harder to manage in Hong Kong.’* *‘They have a variety of resources – different educational materials and databases. Here in Indonesia, I think we only have materials from the Health Ministry. When the Health Ministry says something, we always follow along. But they have more than that, and this information is helpful to our project.’ (IN-GP1)* *‘We read each other’s Instagram stories. And I can see that from her story, she was hanging out with her girlfriends and her boyfriend. This is a good exchange. You can learn about their lifestyle, just like health. It really made me become more curious about the Hong Kong culture.’ (IN-GP1)* *‘I gained more understanding of the health situation in another country, and we learned to view health problems not just from a Hong Konger’s perspective. I have learned to care about the health issues of other people around the world. Some places require attention from us.’ (HK-GP1)* *‘It widens our horizons and allows us to understand that everyone is different. At some point, I realized that we have an obligation to help them. We should respect them. You gained insignificant insights and thoughts.’ (IN-GP2)*
**Learning outcome - Adoption of new learning styles from students of other countries**	The quantitative findings explain the qualitative findings. The awareness of cultural difference in learning style allows students to adopt a new learning style from students of other countries. (Convergent)	After the IAH programme, students started to aware more about the impact of cultural value on belief, attitudes and behaviors.	Significant increase in CAS score on subscale ‘cognitive awareness’	**Adoption of new learning styles from students of other countries**	*‘I am only a year 1 student. The Indonesian students were able to think of more aspects and perspectives than me. I learned to view things in a different perspective.’ (HK-GP1)* *‘The first thing I noticed is that students from Indonesia really took the initiative to speak up, and they are more proactive than our Hong Kong students, so I started to be more proactive as well in the discussions.’ (HK-GP1)* *‘I have learned the importance of a team in the two-day workshop on internationalization.’ (INDO-GP1)*
**Learning outcome - Developed cultural sensitivity in clinical practice**	Quantitative and qualitative findings showed consistent findings. The verbatims of qualitative results provide more examples of how students cultural sensitivity in clinical practice had been improved. (Convergent)	Students developed more sensitivity about culture-related clinical issues	Significant increase in CAS score on subscale ‘clinical issues’	**Developed cultural sensitivity in clinical practice**	*‘It widened my horizons. It widened my understanding. It turns out that their practices are different from Indonesians, so for me it’s more.... In the future, if I have to meet patients who I want to interact with, I am going to understand their cultural background first, how to respect, to understand their culture and also their habits to educate them and give them the appropriate intervention.’ (IN-GP2)* *‘It was fun to share something about our habits and cultures. In the group discussions, we got to know about the lifestyle differences between the two countries. Our plan was to create a change in lifestyle for both Hong Kongers and Indonesians. We discussed what kinds of adjustments were needed there, how we should adapt. It was fun!’ (INDO-GP1)*
**Outcome - Improved language skills**	Qualitative findings reveal a theme that is not covered in the quantitative findings. (Divergent)	/	/	**Improved language skills**	*Many of our local students communicate in Cantonese, but in order for us to interact with the Indonesian students, we cannot use our mother tongue. Our English level may not be good enough. I mean, when you want to express your ideas, you may not be able to articulate well. In the end, we have to use English to explain ourselves. I was able to practise my spoken English. (HK-GP1)* *‘It is very interesting, speaking English, listening, and learning, and again to talk to foreigners. Yes, in my opinion I gained additional knowledge.’ (INDO-GP1)*
**Barriers - Poor time management**	Inconsistent to the positive findings of quantitative results, qualitative findings reveal some dissatisfactions related to the IAH program (poor time management). This suggests potential barriers in IAH which may hinder students learning. (Divergent)	Students become more satisfied about the multicultural learning in the university after the IAH program	Significant increase in CAS score on ‘general education and research experience ‘	**Poor time management**	*Personally, it has something to do with the time, the project time. I think the time is too short to work on a project between students of two countries.’ (IN-GP2)* *‘This workshop should not be placed in the senior year, as it clashes with our thesis and community services…. If it is possible, it should be placed in the second or third year.’ (INDO-GP1)*
**Barriers - Insufficient briefing prior to the session**	Inconsistent to the positive findings of quantitative results, qualitative findings reveal some dissatisfactions related to the IAH program (insufficient briefing). This suggests potential barriers in IAH which may hinder students learning. (Divergent)	After the IAH program, Students find the university places more focus on multicultural issues	Significant increase in CAS score on ‘general education and research experience’	***Insufficient briefing prior to the session***	*‘I thought it was a student exchange programme. But that was different from my initial expectation. It turned out to be a programme for learning each other’s culture.’ (IN-GP1)* *‘The overview for this event was a bit sudden (referring to the overview note that the team sent to students prior to the workshop). We didn’t know what this event was about.… The downside would be the short notice beforehand.’ (IN-GP1)*

Remarks: The joint display shows qualitative and qualitative data as well as inferences of the two types of data. The column on meta-inferences are interpretations the research team have drawn after data integration (synthesized area of divergencies and convergencies of both types of data)

## Discussions

Through an analysis of the quantitative data and qualitative data, it was found in this mixed-methods study that the IAH programme is effective at improving cultural awareness. Several themes were identified: (1) learning process: enjoyable, but a desire remains for face-to-face cross-cultural communication; (2) learning outcomes: gained cultural awareness, developed cultural sensitivity, had an opportunity to practise language and learn about new learning styles; (3) factors influencing learning outcomes: facilitators (micro-movie and active communication) and barriers (language barrier, inappropriate time arrangement, insufficient prior briefing).

Similar to our study, Leung et al. [9] found that a five-webinar international programme also led to improvements in the overall cultural awareness of postgraduate students, as well as in two dimensions (general educational & research experience and cognitive awareness). The small sample size (only 18) could explain the lack of change in some dimensions. However, Psychouli et al. [26] suggested that there was no change in the cultural awareness of students after an international online conferencing collaboration and that the students had a strong desire to participate in more frequent and culturally diverse experiences.

We were able to gain a further understanding of the students’ feelings, what they had learned, and their opinion of this programme by holding focus groups to provide some valuable suggestions on future IAH programmes. Three themes were identified, of which the first concerned the learning process. Our study demonstrated that most students recognized that IAH was novel and enjoyable, but they still desired face-to-face cross-cultural communications. A large investigation involving 213,160 undergraduate students from nine universities indicated that undergraduate students who participated in IAH activities reported higher levels of cultural awareness compared with those who had studied abroad [27]. Therefore, they concluded that IAH was just as effective as, if not more effective than, formal study abroad [27]. This might be because when students study abroad, they are in a new environment and normally experience anxiety, which has the potential to negatively influence their communications with others; while when they are in a familiar environment (i.e., their own home, school), they are much more comfortable interacting with people from another culture [28]. Besides, not all students are able to study abroad because of financial issues and limited time. One study found that at most 10% of students were able to engage in mobility programmes abroad [29].

With regard to the learning outcomes, our study found that students gained new knowledge and increased levels of cultural awareness, which further validated the results of the quantitative data. Noticing cultural differences between the two countries often came as a surprise to students, indicating that they might have had few chances in the past to participate in inter-cultural activities or to meet people from different cultural backgrounds, whether online or offline, which was also found in other study [30]. Thus, it is essential to have cross-cultural activities or programmes at school or to integrate IAH in the curriculum, so that international programmes can be available to all students in their home campus [26]. In addition to new knowledge, a few students in this study developed a sense of international social responsibility and cultural sensitivity in clinical practice. IAH not only has the potential to prepare students for clinical practice; more importantly, it generates in them a sense of civic responsibility in a global world. COVID-19, as well as other global health events, such as outbreaks of the Ebola virus and SARS, all indicate the need for international communication and collaboration among health workers around the world [10]. Therefore, a sense of international responsibility is needed today more than ever. Our study showed that participation in a four-session IAH programme can potentially develop a sense of international responsibility in undergraduate students, which is similar to the finding of another study [28].

The last theme was about the factors influencing learning outcomes. We found that a micro-movie and active communication were facilitators of the IAH programme. These two factors in fact make the IAH programme a combination of a case-based study and a cross-cultural one, leading to a high level of realism. The student-led approach encouraged the students to positively communicate with and learn from each other. Similarly, a systematic review indicated that a specially designed case study can improve the cultural awareness and competence of nursing students at a low cost [31]. Some students pointed that more focus can be given in the case to the Indonesian culture. This is a valuable suggestion, which will allow us to further improve the IAH programme. Specifically, we need to be alert to placing equal importance on different cultures in future IAH programmes. On the other hand, there were some barriers to the use of the programme, and language was the one mentioned most often, as in other studies [26, 28, 32]. Language barriers significantly affect communication because people who come from various cultural backgrounds do indeed need more time to process a foreign language to communicate effectively than those from the same cultural background [33]. In addition, language barriers may restrict the depth of focus groups if they are conducted in English or another language that is not the mother tongue of the students [28]. As suggested by our students and by the results of another study [34], smaller groups (*n* < 5) may be a solution to the problems posed by language barriers. The time demands of the IAH programme were also not very suitable to the Indonesian students because they were in their final year and might not have had enough time for the programme. This implies that the IAH programme should be tailored to the participants in order to maximize the benefits to be derived from it. In addition, there were some disagreements among the students in our study on the duration of the activities. One study showed that having activities last less than 1 h at a time is the most effective duration [26], an issue that requires further study.

Overall, we found that our quantitative and qualitative data mostly converged. Both types of data suggested that the major learning outcome of IAH, the enhancement of cultural awareness, was achieved. Our efforts to triangulate the results from two sources of data further enhanced the validity of such findings. The aspects in which there was a divergence between the two sources of data mainly reflect the additional benefits derived from the IAH programme (other than cultural awareness) and logistics concerns (e.g., time management, insufficient briefing), which our quantitative arm had failed to measure. In this study, we only included cultural awareness as the outcome of the quantitative arm. In fact, the students stated that there were several beneficial effects of the IAH programme, such as enhanced social responsibility and language ability. A future study should include other potential outcomes in the measurement. Also, an interdisciplinary approach is advocated nowadays for solving different complicated health problems. In this study, we only included medical and nursing students in the IAH programme. Consideration may be given in a future study to adopting an interdisciplinary approach in the IAH programme to enhance the learning experience of students.

To conclude, this study found that a four-session online internationalization at home programme is effective at improving the cultural awareness of students, as validated by the qualitative data. Moreover, this programme is enjoyable and has the potential to improve the cultural sensitivity of students in clinical practice and their sense of responsibility in a global world. A micro-movie and active communication were facilitators of the programme, while language barriers, time management, and insufficient prior briefing were barriers, which should be addressed or avoided in future studies.

### Strengths and limitations

First, this is one of the very few studies to explore students’ experience of an IAH programme during a pandemic. It provides a unique understanding of how IAH could work when all face-to-face cultural exchange sessions are suspended. Also, our IAH programme was co-designed by medical and nursing professionals from both of the countries involved. Such a collaboration can make the programme a good fit for the needs of medical and nursing students from both countries. More importantly, the method employed in our study was a rigorous one, and triangulation was used to verify the quantitative and qualitative data. This method also provided a broader picture and a more in-depth understanding of the learning experience of students than did previous studies. However, this study has its limitations. The participants were all students who had chosen to enrol in the course. They were likely to be those who enjoy cultural exchange activities. Our findings may not be fully generalizable to all students in universities in Hong Kong and Indonesia. Also, all of our focus groups were conducted by the instructors of the course. This could have affected the equality of the relationship between the instructors and the participants (raising hierarchy issues). The participants might have felt compelled to provide more desirable feedback in the focus groups than they would otherwise have given.

## Supplementary Information

**Additional file 1: Supplementary Table 1.** Legitimation criteria in this study.

## Data Availability

The datasets generated and/or analysed during the current study are not publicly available due to an agreement with the subjects but are available from the corresponding author on reasonable request.
