# Supplementary material for: Effects of an internationalization at home (IAH) programme on cultural awareness among medical and nursing students in Hong Kong and Indonesia during the COVID-19 pandemic: a mixed-methods study
Source: BMC Med Educ. 2022 May 13;22:368. doi: 10.1186/s12909-022-03424-5 (PMC9099034; doi:10.1186/s12909-022-03424-5)
Supplement: Supplementary file 1 — Additional file 1: Supplementary Table 1. Legitimation criteria in this study. [file 12909_2022_3424_MOESM1_ESM.docx]

Additional file 1. Supplementary Table 1

| **Supplementary Table 1. Legitimation criteria in this study.** | |
| --- | --- |
| **Legitimation type** | **Strategies used** |
| Commensurability approximation legitimation | The research was conducted by experienced researchers in qualitative, quantitative, and mixed methods.  The qualitative approach was used to investigate the emic perspective, while the quantitative methodology focused on etic views. The involvement of an expert in mixed methods ensured a balanced account of both views. |
| Inside-outside legitimation | Quantitative data and quotes from the qualitative phase are summarized in the Joint Display (Table 3).  To explore the emic perspective (students’ views), a thematic analysis was used.  To ensure inter-rater reliability, two researchers independently coded the texts of the focus groups.  To maintain a balance between emic and etic views, the researchers analysed qualitative and quantitative data together. |
| Integration legitimation | In all phases of the study, the authors tried to integrate the quantitative and the qualitative data. This is shown in the Joint Display (Table 3). |
| Paradigmatic legitimation | The research questions guided the researchers in choosing the most suitable methodologies to decide the phases of the study, as well as the methods of collecting and analysing the data.  We asked ourselves the following questions:   - Should we use a validated questionnaire to assess cultural awareness? - Should we use content or thematic analysis?   The research team agreed to use a validated scale to assess the cultural awareness of the participants and to adopt a thematic analysis to understand the perceptions of the patients and facilitators, as well as the barriers to the use of the programme. |
| Sample integration legitimation | With regard to the quantitative data, we used a convenience sample, which was not representative. Our intent was not to achieve generalizability, but to evaluate these variables in our context.  For the qualitative phase, the sample was purposive and the variability was reasonable. |
| Sequential legitimation | The research team agreed to use a convergent mixed-methods design because both types of data have equal value for understanding  the research questions. |
| Socio-political legitimation | The results of our study highlighted the usefulness of the IAH programme, as well as the facilitators and barriers to the use of the programme.  This study offers useful information for healthcare educators and other stakeholders to conduct IAH programmes in the future to increase the cultural awareness of students. |
| Weakness minimization legitimation | For the quantitative data we used a validated tool (CAS). For the qualitative data we used Guba and Lincoln’s criteria, and for data integration we used the joint display.  Through these methods we tried to minimize the limits of each approach, trying to achieve the added value of gaining further insight into the problem, which would have been difficult to achieve if two separate approaches had been used. |
